# Factors associated with mental health of internal migrants (Kayayei) in Agbogbloshie-Ghana

**DOI:** 10.1186/s12905-023-02582-y

**Published:** 2023-08-25

**Authors:** Joyce komesuor, Anna Meyer-Weitz

**Affiliations:** 1 School of Public Health, University of Health and Allied Sciences, Ho, Ghana; 2 School of Applied Human Sciences, University of KwaZulu-Natal, Durban, South Africa

**Keywords:** Mental health, Kayayei, Internal migrants

## Abstract

**Background:**

The United Nations (UN) Sustainable Development Goal (SDG) Eight (8) advocates for decent work and improved economic outcomes for all. However, internal migrant workers in Ghana, especially female head porters, commonly known as “Kayayei”, work in exploitative and hazardous conditions exposing them to physical and mental health risks. Yet, mental health among this vulnerable group of migrants has not been given the needed attention it deserves in the country. We, therefore, examined the factors associated with mental health challenges among internal migrants (Kayayei) in Ghana.

**Methods:**

A cross-sectional study among a systematic random sample of 352 Kayayei was conducted in Agbogbloshie-Accra, Ghana. An interviewer-administered questionnaire was used to collect data on the factors impacting the mental health of Kayayei. The study used binary logistic regression in predicting factors impacting mental health distress at a 0.05 level of significance and 95% confidence interval.

**Results:**

The prevalence of depression, anxiety, and stress were, 305 (86.6%), 332 (94.4), and 149 (42.4), respectively, with 147(41.1%) of respondents having all three mental health issues. The difficult nature of work significantly predicted depression, anxiety, and stress. Respondents who perceived their work as very difficult were 4.3 times, (aOR = 4.36, 95% CI = 2.17, 8.76, *p* =  < 0.001), 3.66 times (aOR = 3.66, 95% CI = 1.37, 9.76, *p* = 0.009), and 1.73 times (aOR, = 1.73, 95% CI = 1.04, 2.85, *p* =  < 0.009) more likely to be depressed, anxious, and stressed respectfully as compared to those who rate their work as just difficult**.**

**Conclusion:**

The majority of the Kayayei suffered from mental health distress (depression, anxiety, and Stress) due to their work circumstances. This study suggests that the Ghana Labour Commission must extend the Labour Act 2003(Act 651) to cover the informal sector and create awareness among the Kayayei community to know their rights and report any abuse to law enforcement agencies. It is also suggested that the government, NGOs, and other benevolent organisations train the Kayayei to attain alternative and sustained livelihoods that will not negatively impact their mental health as has been found in the current study. Finally, the government should fully implement the 2012 Mental Health Act to increase awareness and access to quality mental health care**.**

## Introduction

The United Nations (UN) Sustainable Development Goal (SDG) Eight (8) advocates for decent work and improved economic outcomes for all [1]. However, migrant workers, particularly women, work in exploitative and hazardous conditions exposing them to physical and mental health risks [2]. In Ghana, like many low- and middle-income countries in sub-Saharan Africa, an uneven distribution of resources and economic opportunities serve as a migration-push factor to resource-rich areas in the southern part of the country, specifically Accra, the capital city of Ghana, in search of better living conditions [3]. The north–south migration offers the opportunity for remittances that migrants send home as a source of income to many households in northern Ghana, particularly during economic shock [4, 5]. Therefore, the youth from the north venture on a southward migration journey to get employment to earn income as well as support their relations back home [6]. However, there is an underestimation of poverty levels in large cities in terms of paying for food, water, accommodation, and transport, which are free in rural areas [7]. Migrants, therefore, in trying to escape poverty from the rural areas, seem rather to transport the poverty from the place of origin to the areas of destination [8].

Kayayei or Kaya Yei is a Ghanaian term that refers to a female head porter or bearer [9]. They usually operate at market centres and lorry stations carrying goods on their head for shoppers [10]. Head porterage is one of Ghana's main forms of transport of goods. People bring their wares from farms to their homes on their heads [11]. Many Kayayei usually settle in slums in Accra, where other migrants are located [12]. The areas are, however, densely populated and lack basic facilities, including health care services [10, 13].

There is ongoing theoretical debate on developing mental health challenges due to international migration, social and environmental factors such as traumatic events, daily stressors, adversity, and chronic strain as having adverse impacts on mental health [14–16]. Studies on the effects of internal migration on mental health have produced diverging and sometimes contradictory results [17–21]. For instance, a previous study by Switek [21] among young adults in Sweden found that internal migration is accompanied by increased life satisfaction due to increased income levels. A related study in China [22] found that migrant workers reported a lower prevalence of depression than non-migrant workers. In another study in Indonesia, by contrast, the results show that migrants tended to have mental health challenges such as depression [23]. Notwithstanding these contradictions in the literature, there is no doubt that migrants are exposed to different forms of mental health challenges.

Whilst migrants are usually faced with physical and mental health issues, the focus of various researchers has been mostly on the flow of remittances and the direction of migration, thus from rural areas or smaller towns to cities [8]. Migration subsequently may result in challenges, such as unemployment, poverty, and the development of slum communities [8]. Furthermore, physical and mental health issues have not been given the necessary attention they deserve in Ghana. For instance, in a systematic review of mental health research [24], the researchers noted that between 1955 and 2009, 98 articles were published in peer-reviewed journals on mental health in Ghana. Several of these studies were on drug use, with only one on homelessness (due to poverty) and mental health. For instance, a study was conducted on the socio-demographic characteristics of substance abusers admitted to a private specialist clinic [25], while another study [26] investigated the quality of psychotropic drug prescriptions at Accra Psychiatric Hospital. On homelessness, another study [27] was conducted on homelessness and mental health in Ghana and explored the lived experiences of squatters in settlements in East-Legon, an affluent neighbourhood in Accra. This shows that there is paucity of data on the topic under study. This study, therefore, sought to bridge the literature gap by determining factors associated with the mental health of internal migrants (Kayayei) in Agbogbloshie-Ghana, one of the country's largest and busiest markets. The study hypothesized that the working conditions of Kayayei will have a negative impact on their mental health. Understanding the factors that impact the mental health of the Kayayei could be essential for developing intervention strategies to promote the mental well-being of such vulnerable groups in Ghana and also accelerate the progress towards achieving SDG 3.

## Methods

### Study design and period

This was a descriptive cross-sectional study where samples were drawn from the population at one point in time [28]. Data were collected from August to September 2018. Data was collected through an interviewer-administered questionnaire using English, Twi, Mamprusi, and Silasa. The questionnaire consisted of two broad sections; sections A and B. Section A had questions on the background characteristics of respondents, and section B was on the mental health status (depression, anxiety and stress) of respondents.

### Study population

All Kayayei from the three northern regions of Ghana who plied their trade in the Agbogbloshie market constituted the population for the study. The total population of Kayayei in Agbogbloshie (total registered by the Kayayei Association) market is about 3000 [29]. To be included in the study, participants had to be a Kayayo, be 18 years and above, must have been in Accra for more than six months, should have come from one of the three northern regions and was willing to give informed consent.

### Sample size determination and sampling

The sample size was determined based on the study population, thus, a population of 3000 Kayayei at the market, as recommended by Krejcie and Morgan [30]. Hence, the computed sample size was 375 participants after adjusting for a ten percent non-response rate. Multi-stage sampling strategy was used for the study. First, convenient sampling was used to select the Agbogbloshie market because it is one of the largest and busiest markets in the country, with a lot of Kayayei activities [31]. Second, stratified sampling was used to divide the market into two strata: the 'main market' and the 'yam market'. Since the proportion of Kayayei operating in both markets was unknown, the study assumed a conservative distribution of 50% representation at both sub-markets. After this, a systematic sampling procedure was used to select the respondents. The research team went to a vantage point at the market (the main entrance to the market) and the first Kayayei who was approached and agreed to participate in the study was indexed as 1 and engaged. Subsequently, every eighth person was recruited to participate in the study. In situations where a respondent refused to partake in the study, the next consenting Kayayei was recruited.

### Measures

#### Independent variables

The bio-demographic data were collected and used to understand the characteristics of the target group. These include questions relating to age, education, marital status, number of children, religion, duration of stay in Accra, accommodation, daily work duration, daily income, and treatment by clients (Table 1).

**Table 1:** Study variables

Variables	Question	Response options and recoding
**Dependent variables**		
**The DASS-21 scale:** All items on the scale were assessed as;		
0 “Did not apply to me at all”		
1 “Applied to me to some degree, or some of the time”		
2 “Applied to me to a considerable degree or a good part of time”		
3 “Applied to me very much or most of the time”		
**Coded as**		
**0 “Did not apply to me at all”**		
**1 “Applied to me to some degree, or some of the time”**		
**2 “Applied to me to a considerable degree or some of the time”**		
**3 “Applied to me very much or most of the time”**		
Depression	I couldn't seem to experience any positive feelings at all	
	I found it difficult to work up the initiative things to do	
	I felt that I had nothing to look forward to	
	I felt down-hearted and blue	
	I was unable to become enthusiastic about anything	
	I felt I wasn't worth much as a person	
	I felt that life was meaningless	
Stress	I found it hard to wind down	
	I tended to over-reach to situations	
	I felt that I was using a lot of nervous energy	
	I found myself getting agitated	
	I found it difficult to relax	
	I was intolerant of anything that kept me from getting on with what I was doing	
	I felt that I was rather touchy	
Anxiety	I was aware of dryness of my mouth	
	I experienced breathing difficulty (e.g. excessively rapid breathing, breathlessness in the absence of physical exertion)	
	I experienced trembling (e.g. in the hands)	
	I was warried about situations in which I might panic and make a fool of myself	
	I felt I was close to panic	
	I was aware of the action of my heart in the absence of physical exertion (e.g. sense of heart rate increase, heart missing a beat)	
	I felt scared without any good reason	
**Independent variables**		
Age	How old are you??	Open question **(coded as 18–24 = 1, 25–34 = 2, ≥ 35 = 3)**
Education	What is your educational Status	1 = None, 2 = Primary, 3 = JHS, 4 = SHS
Marital Status	What is your marital status?	1 = married. 2 = Unmarried
Religion	What is your religion?	1 = Christianity, 2 = Islam
Duration of stay in Accra	How long have you been in Accra?	1 = < 1, 2 = 1–2,3 = ≥ 3
Accomodation	Do you have accomodation?	1 = No, 2 = Yes
During of work in a day	How many hours do you work in a day?	1 = 1–8 h, 2 = 9 h or more
Daily Income	How much do you earn in a day? In Ghana cedis	Open question 1 = 1–10, 2 = 11–20,3 = ≥ 21
Difficulty of work	How do you rate work work?	1 = difficult, 2 = very difficult
Treatment by customers	How do your customers treat you?	1 = nicely, 2, normal, 3 = badly
Number of children	How many children do you have?	1 = none, 2 = 1-2, 3 = ≥3

#### Dependent variables

Depression, Anxiety, and Stress Scale (DASS-21): Mental health challenges were measured using the DASS 21 scale [32]. The DASS-21 is made up of a set of three self-report scales intended to assess the emotional states of depression, anxiety and stress. Each of these DASS-21 scales contains 7 items; the items are divided into subscales with similar content. The depression scale measures dysphoria, hopelessness, devaluation of life, self-deprecation, lack of interest / involvement, anhedonia, and inertia. The anxiety scale measures autonomic arousal, skeletal muscle effects, situational anxiety, and subjective experience of anxious affect. The stress scale assesses difficulty relaxing, nervous arousal and being easily upset/agitated, irritable/over-reactive and impatient [32]. The DASS 21 is rated on a 4-point Likert scale (Did not apply to me, applied to me to some degree, or some of the time, applied to me to a considerable degree or a good part of time, and applied to me very much or most of the time) (Table 1). The total score for each sub-scale is multiplied by 2 to get the final score. Scores for depression, anxiety and stress were calculated by summing the scores for the relevant items. The score for depression ranged from (0–9 for normal; 10–13 for mild; 14–20 for moderate; 21–27 for severe and 28 + for extremely severe depression. The score for anxiety ranged from (0–7 normal, 8–9 mild, 10–14 moderate, 15–19 severe, and 20 + extremely severe, finally the score for stress also ranged from (0–14 normal, 15–18 mild, 19–25 moderate, 26–33 severe, and 34 + extremely severe) [32]. The DASS-21 showed high internal consistency in the African context [33]. The present study's Cronbach alpha (α) for the overall scale was (0.93), and the sub-scales of depression, anxiety and stress were 0.85, 0.80, and 0.77, respectively (Table 4).

### Procedure

Ethical clearance was obtained from the College of Humanities, University of KwaZulu Natal Research Ethics Committee in South Africa (Ref: HSS/0404/018D). To conduct a study among the Kayayei, it was required to obtain permission from the Kayayei Association in Accra. The chairman of the Kayayei Association was consulted, and written permission was obtained to conduct the study among the members. After obtaining permission from the association, a memorandum of understanding was signed between the Kayayei Association and the researchers to conduct the study. Two research assistants were trained on the data collection instruments and ethics of data collection as well as on how to collect the data. Two undergraduate students, fluent in Twi, Mamprusi, and Sisala, the dominant languages among the Kayayei population were also trained to translate the instruments to respondents who did not understand English. The study team convened prior to data collection to practice the procedure and go over the best way to translate keywords into the local languages while maintaining consistency in translation. Respondents were briefed about the aim and objectives of the study and those willing to take part gave informed consent by signing or thumb-printing the consent form. Data collection was strictly supervised by the first author. It took an average of 30 min to administer the full questionnaire. Data collection lasted for two months.

### Data analyses

Data were collected using CSpro software and later exported to Stata 15 for data analysis. The data collected were subjected to a thorough quality control process to ensure that the data were as accurate and complete as possible. Descriptive statistics on both continuous and categorical variables were obtained using Stata 15. A descriptive analysis for plausibility checks was performed to address any inconsistencies. Furthermore, minimum and maximum scores were generated for each item to ensure that all the measures were within the expected range of the possible score [34]. This helped in identifying missing values and errors and in cleaning duplicates. The psychometric properties of all the measures and sub-scales were determined using inter-item reliability coefficients, i.e., Cronbach's alpha. In this study, the assumption of normality of the scores and homogeneity of variances were tested. The Shapiro–Wilk test for normality was conducted to examine the normality of all measures. The results indicated most of the scores were fairly normal (*p* > 0.05). Levene's test for homogeneity of variance did not reveal much variance in the score of the various measures. Variance Inflation Factor (VIF) was used to check for multicollinearity which revealed that the independent variables were not collinear (Table 2). Frequencies and percentages were used to describe the independent variables (age, education, marital status, number of children, religion, duration of stay in Accra, accommodation, daily work duration, daily income, work difficulty and treatment by clients). The prevalence of mental health distress was estimated based on the DASS-21 classification [32]. The DASS-21 scale was re-coded as psychological normal and psychological distressed [35–37]. The respondents with scores in the normal and mild range were classified as psychological normal, while those with scores in the moderate to extremely severe range were classified as psychological distressed. Univariate and multivariate binary logistic regressions were performed to determine the predictors of mental health challenges. Binary logistic regression was performed to predict the likelihood of participants being psychological distressed based on their background characteristics. In model one, we quantified the association between background characteristics and individual mental health challenges by calculating the Odds ratios (ORs) with 95% Confidence Intervals (CIs). We use forward stepwise selection to produce a final model to determine the statistically significant variables affecting particular mental health distress (Depression, Anxiety, and Stress) at a probability value of 0.50. The results were presented as adjusted Odds Ratio (aOR) with *p*-value less than 0.05 considered statistically significant at 95% confidence interval (CI).

**Table 2:** Variance inflation factor estimation

Variable	VIF	1/VIF
Number of Children	2.62	0.381977
Marriage	2.06	0.484768
Age	1.92	0.519776
Education	1.53	0.654818
Years in Accra	1.49	0.671922
Work Duration	1.44	0.694304
Daily Income (GHC)	1.39	0.716975
Treatment by clients	1.27	0.785354
Accommodation	1.25	0.80305
Work Difficulty	1.06	0.946735

## Results

### Background characteristics of participants

Table 3 presents the background characteristics of Kayayei. The median age of the study participants was 25 (IQR = 18–65). Majority (47.4%) were between 18–24 years old and 38.6% had no formal education. Most (63.1%) were married and 40.5 per cent had given birth to one to two children. The Kayayei primarily belonged to the Islamic religion (86.7%,). The results also indicated that 36.9 percent had been living in the city for at least three years. The results further indicate that majority of the Kayayei (66.8%) rated their work as very difficult. Majority also indicated having accommodation (51%) in the city. Most (52.4%) of the Kayayei usually worked up to eight hours a day and generally earned GHC 11–20 (54.3%) ($1 = GHC 4.9 [in 2018 when data were collected). The mean income was 14.63 ± 6.78 and median income was GHC 15. Clients, according to majority of the Kayayei, treated them as 'normal' (53.1%).

**Table 3:** Background characteristics of participants

Characteristic	Frequency [*N* = 352]	Percentage [%]
**Age (Years)**		
18–24	167	47.4
25–34	138	39.2
≥ 35	47	13.4
**Median(± SD)**	**25 (18–65)**	
**Education**		
No Education	136	38.6
Primary Education	112	31.7
JHS	83	23.5
SHS	21	6.2
**Marital status**		
Unmarried	130	36.9
Married	222	63.1
**Number of Children**		
No child	95	27.0
1–2 children	143	40.5
≥ 3	115	32.5
**Religion**		
Christianity	50	14.2
Islam	302	85.8
**Duration of stay in Accra (In years)**		
< 1	101	28.7
1–2	121	34.4
≥ 3	130	36.9
**Accommodation in Accra**		
No	172	49.0
Yes	179	51.0
**Daily work duration (In hours)**		
1–8	184	52.4
9 +	167	47.6
**Daily income (GHC)**		
1–10	133	37.8
11–20	191	54.3
≥ 21	28	7.9
Mean(± SD)	14.63 (± 6.78)	
**Difficulty of work**		
Difficult	235	66.8
Very Difficult	117	33.2
**Treatment by clients**		
Nicely	88	25.0
Normal	187	53.1
Badly	77	21.9

### Prevalence of mental health challenges among Kayayei

**Fig. 1:**
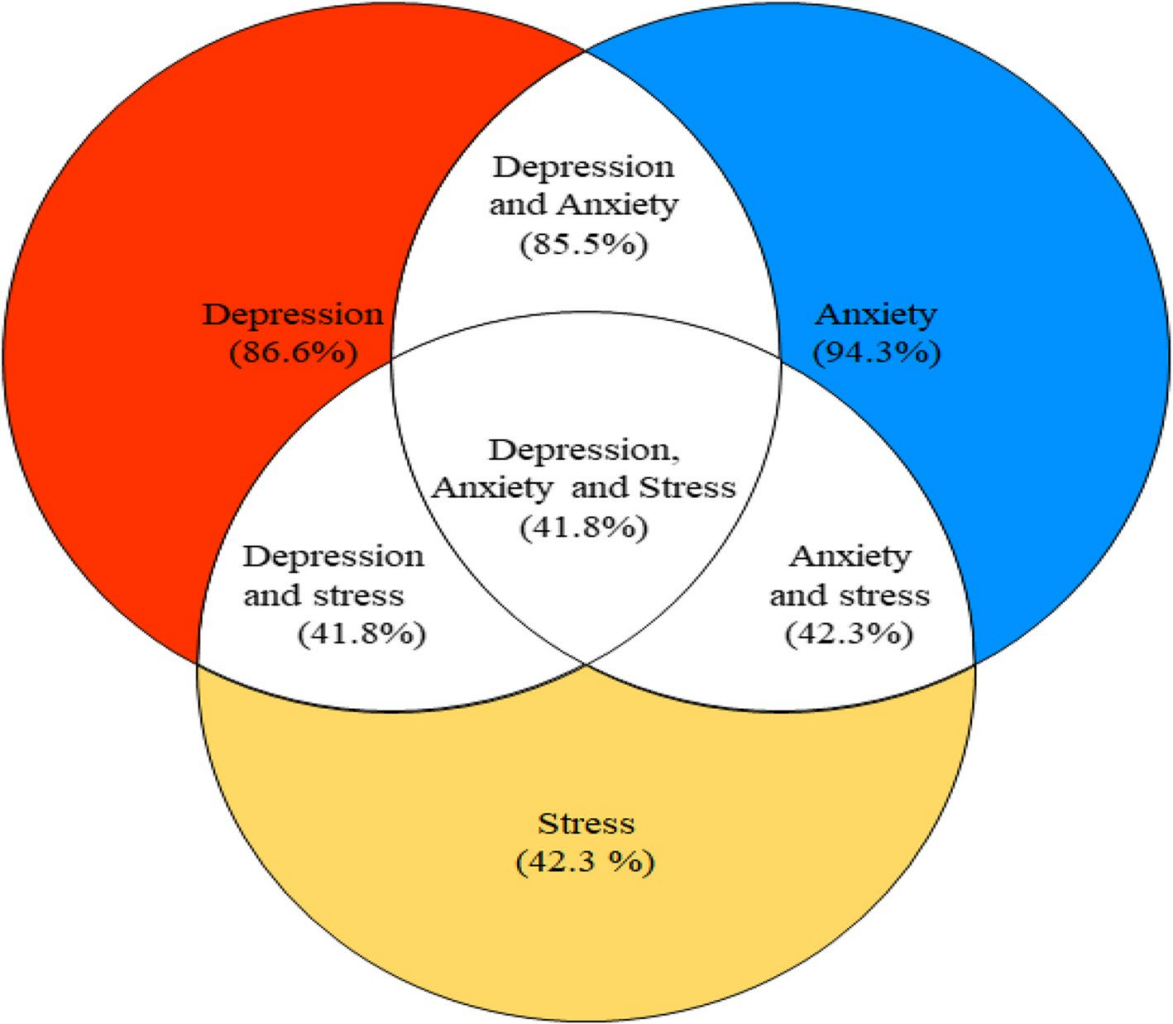
Prevalence of mental health challenges

Figure 1 presents the prevalence of mental health challenges among study respondents. Depression, anxiety and stress among the Kayayei were 86.6%, 94.4%, and 42.4%, respectively. Overall, 41.8% experienced all three mental health challenges in this study. The findings also indicated that simultaneously, 85.8% experienced depression and anxiety alone, 41.8% experienced depression and stress alone, and 42.3% experienced anxiety and stress alone.

### Spearman correlation variables

Table 4 presents correlations among variables. Age is positively correlated with marital status (0.473), number of children (0.738), daily income (0.346), and number of years in Accra (0.464). However, age is negatively correlated with education (-0.425), work difficulty (-0.108), treatment by clients (-0.123) and depression (-0.205). There is also negative correlation between education and marriage (-0.483), number of children (-0.470), daily income (-0.193), and years in Accra (-0.169). On the other hand, education has a positive correlation with work difficulty (0.171), accommodation (0.280), treatment by clients (0.119), depression (0.233), anxiety (0.143), stress (0.143), and overall mental health distress (0.136). Marriage is positively correlated with number of children (0.593), work duration (0.157), daily income (0.230), and years in Accra (0.173). Marriage is however negatively correlated with accommodation (-0.206), and depression (-0.172). The number of children is also positively correlated to daily income (0.286), duration in Accra (0.304), but negatively correlated with depression (-0.118). Work difficulty is positively correlated with accommodation (0.155), and mental health distress (0.258). Accommodation is positive correlated with treatment by clients (0.113) and mental health (0.235). Treatment by clients is negatively correlated by work duration (-0.366), income (-0.147), and duration in Accra (-0.152). It is however, positively correlated with overall mental health (0.138). Work duration is positively correlated to daily income (0.388), and duration in Accra (0.129), but negatively correlated to stress (-0.145), and overall mental health (-0.148) There is a positive correlation between daily income and duration in Accra (0.412), but a negative correlation with depression (-0.245). Years in Accra is negatively correlated with depression (-0.197), and stress (-0.157), and overall mental health (-0.138). Depression is positively correlated with anxiety (0.777), stress (0.793).

**Table 4:** Spearman correlations between variables

Variables	(1)	(2)	(3)	(4)	(6)	(7)	(8)	(9)	(10)	(11)	(12)	(13)	(14)	(15)
**(1) Age**	**1.000**													
**(2) Education**	-0.425***	**1.000**												
**(3) Marital status**	0.473***	-0.483***	**1.000**											
**(4) Number of Children**	0.738***	-0.470***	0.593***	**1.000**										
**(6) Work Difficulty**	-0.108*	0.171***	**-0.053**	**-0.070**	**1.000**									
**(7) Accommodation**	**-0.094**	0.280***	-0.206***	**-0.095**	0.155**	**1.000**								
**(8) Treatment by clients**	-0.123*	0.119*	-0.112*	**0.007**	**0.082**	0.113*	**1.000**							
**(9) Work Duration (hrs)**	**0.028**	**-0.083**	0.157**	**-0.010**	**-0.029**	-0.264***	-0.366***	**1.000**						
**(10) Daily Income (GHC**	0.346***	-0.193***	0.230***	0.286***	**-0.059**	**-0.087**	-0.147**	0.388***	**1.000**					
**(11) Years in Accra**	0.464***	-0.169**	0.173**	0.304***	**-0.013**	**0.003**	-0.152**	0.129*	0.412***	**1.000**				
**(12) Depression**	-0.205***	0.233***	-0.172**	-0.118*	0.320***	0.404***	0.264***	-0.254***	-0.245***	-0.197***	**1.000**			
**(13) Anxiety**	**-0.002**	0.109*	**0.006**	**0.026**	0.250***	0.360***	0.147**	**-0.100**	**-0.025**	**-0.046**	0.777***	**1.000**		
**(14) Stress**	**-0.071**	0.143**	**-0.065**	**-0.042**	0.233***	0.330***	0.107*	-0.145**	**-0.103**	-0.157**	0.793***	0.822***	**1.000**	
**(15) Overall MH**	-0.133*	0.136**	**-0.064**	**-0.098**	0.258***	0.235***	0.138**	-0.148**	-0.119*	-0.138**	0.804***	0.773***	0.821***	**1.000**
											**7(8.85)**	**7(0.80)**	**7(0.77)**	**21(0.93**

^*^ *p* < 0.05, **p < 0.01 and ***p < 0.001; n-number of items measured, α-alpha

### Predictors of mental health challenges among the Kayayei

Table 5 indicated that respondents who perceived their work as very difficult were 4.36 times (aOR = 4.36, 95% CI = 2.17, 8.76, *p* =  < 0.001), 3.66 times (aOR = 3.66, 95% CI = 1.37, 9.76, *p* = 0.009), and 1.73 times (aOR, = 1.73, 95% CI = 1.04, 2.85, *p* =  < 0.009) more likely to be depressed, anxious, and stressed respectfully as compared to those who rate their work as just difficult. Furthermore, participants who perceived to be treated normally by clients were 2.26 times more likely to be depressed as compared to those who perceived being treated nicely (aOR = 2.26, 95% CI = 1.02, 5.02, *p* = 0.046). Concerning education, respondents with primary education were 4.88 (aOR = 4.8, 95% CI = 1.05, 22.74, *p* = 0.043) and 1.81 times (aOR = 1.81, 95% CI = 1.02, 3.19, *p* = 0.042) more likely to be anxious and stressed respectfully as compared to the uneducated. Furthermore, respondents with accommodation were 1.73 times more likely to be stressed (aOR = 1.73 95% CI = 1.04, 2.85, *p* = 0.033) as compared with those without accommodation.

**Table 5:** Predictors of depression, anxiety, and stress

Parameters	Depression		Anxiety		Stress	
	**cOR (95% CI) *p*-Value**	**aOR (95% CI) *p*-Value**	**cOR (95% CI) *p*-Value**	**aOR (95% CI) *p*-Value**	**cOR (95% CI) *p*-Value**	**aOR (95% CI) *p*-Value**
**Age (years)**						
18–24	**Ref**	**Ref**	**Ref**	**Ref**	**Ref**	**Ref**
25–34	1.12 (0.55, 2.27), 0.763	1.49 (0.63, 3.54), 0.366	1.25 (0.43, 3.61), 0.676	1.46 (0.45, 4.80), 0.529	1.30 (0.82, 2.04), 0.264	1.40 (0.80, 2.43), 0.236
≥ 35	0.40 (0.18, 0.89), **0.024**	0.62 (0.23, 1.67), 0.342	0.48 (0.15, 1.50), 0.207	0.68 (0.18, 2.59), 0.578	0.75 (0.38, 1.48), 0.409	1.12 (0.50, 2.53), 0.780
**Education**						
Not Educated	Ref	Ref	Ref	Ref	Ref	Ref
Primary	2.64 (1.22, 5.72), **0.013**	1.92 (0.82, 4.48), 0.134	5.81 (1.28, 26.33), **0.022**	4.88 (1.05, 22.74), **0.043**	2.33 (1.39, 3.90), **0.001**	1.81 (1.02, 3.19), **0.042**
JHS	2.81 (1.17, 6.78), **0.021**	1.37 (0.47, 4.05), 0.563	2.82 (0.78, 10.20), 0.114	2.27 (0.52, 9.81), 0.272	1.76 (1.01, 3.10), **0.047**	1.17 (0.59, 2.32), 0.642
SHS	2.46 (0.54, 11.21), 0.244	1.24 (0.22, 7.17), 0.808	1.00 (0.21, 4.80), 0.996	0.69 (0.12, 3.91), 0.675	1.29 (0.50, 3.33), 0.603	0.67 (0.23, 1.94), 0.462
**Marital Status**						
Unmarried	Ref		Ref		Ref	
Married	0.54 (0.27, 1.09), 0.085		1.15 (0.46, 5.88), 0.772		0.82 (0.53, 1.27), 0.375	
**Number of Children**						
No child	Ref		Ref		Ref	
1–2	1.31 (0.56, 3.06), 0.533		4.75 (0.94, 24.07), 0.060		0.80 (0.47, 1.35), 0.409	
3 and above	0.52 (0.24, 1.13), 0.097		0.57 (0.21, 1.59), 0.285		0.91 (0.53, 1.58), 0.741	
**Work Difficulty**						
Difficult	Ref	Ref	Ref	Ref	Ref	Ref
Very Difficult	4.42 (2.32, 8.43), < **0.001**	4.36 (2.17, 8.76), < **0.001**	4.07 (1.58, 10.50), **0.004**	3.66 (1.37, 9.76), **0.009**	2.08 (1.30,3.32), 0.002	1.73 (1.04, 2.85), **0.033**
**Accommodation**						
No	Ref	Ref	Ref		Ref	Ref
Yes	2.02 (1.07, 3.83), < **0.001**	0.91 (0.41, 2.03), 0.820	1.05 (0.42, 2.59), 0.917		3.48 (2.23,5.44), < **0.001**	2.91 (1.71, 4.98), < **0.001**
**Treatment by clients**						
Nicely	Ref	Ref	Ref		Ref	Ref
Normal	3.75 (1.89, 7.44), < **0.001**	2.26 (1.02, 5.02), **0.046**	2.22 (0.75, 6.54), 0.147		2.31 (1.34, 3.96), **0.002**	1.23 (0.63, 2.39), 0.543
Badly	4.44 (1.71, 11.55), **0.002**	2.67 (0.83, 8.54), 0.098	1.02 (0.33, 3.18), 0.969		1.61 (0.84, 3.06), 0.150	1.03 (0.47, 2.24), 0.944
**Work Duration (hrs)**						
1–8	Ref	Ref	Ref		Ref	Ref
9 and above	0.35 (0.18, 0.68), **0.002**	0.51 (0.21, 1.22), 0.128	0.81 (0.32, 2.04), 0.651		0.63 (0.41, 0.96), **0.033**	1.03 (0.59, 1.80), 0.917
**Daily Income (GHC)**						
1–10	Ref	Ref	Ref		Ref	Ref
11–20	0.34 (0.15, 0.72), **0.005**	0.69 (0.27, 1.73), 0.430	0.58 (0.20, 1.69), 0.321		0.62 (0.39, 0.97), **0.036**	0.75 (0.42, 1.32), 0.317
≥ 21	0.44 (0.12, 1.53), 0.195	0.82 (0.17, 4.06), 0.813	0.32 (0.07, 1.45), 0.141		0.91 (0.40, 2.05), 0.814	0.76 (0.30, 1.93), 0.566
**Duration in Accra**						
< 1	Ref		Ref		Ref	
1–2	1.35 (0.57, 3.20), 0.498		2.86 (0.85, 9.59), 0.088		0.80 (0.47, 1.36), 0.409	
≥ 3	0.59 (0.28, 1.26), 0.174		1.72 (0.62, 4.79), 0.300		0.62 (0.37, 1.05), 0.077	

*χ2* Chi Square, *CI* Confidence interval, *cOR* Crude odds ratio, *aOR* Adjusted odds ratio

## Discussion

This cross-section study examined factors associated with the mental health of 352 internal migrants (Kayayei) in Agbogbloshie-Ghana. The study hypothesised that the working conditions of the Kayayei will have negative impact on their mental health. The study found that 41,8%, of respondents have all three mental health issues (depression, anxiety, and stress) which is higher than those observed by Amu et al. (8.3%) [38] and Sweetland et al. (30.4%) [39]. The differences may be attributed to the different populations. The present study was among low-income highly distressed population who work in hazardous situations. The findings of this present study showed a comparatively lower prevalence of stress (42.3) in comparison to the extremely high prevalence of anxiety (94.3) and depression (86.6) among the Kayayei. This finding contradicts previous findings that observed a higher prevalence of stress rather than anxiety and depression among migrants [40, 41]; the present study's deviation may be due to the unique background context of the Kayayei. Before migration, these women often had menial jobs on farms to support themselves and their families including having domestic duties [42, 43]. These Kayayei are therefore used to working under challenging conditions which might account for the low levels of stress recorded in the present study. The high prevalence of mental health distress among the Kayayei population indicates that not much progress has been made in attaining SDG 3–4, particularly in promoting mental health and well-being for all by 2030. To promote mental health for all, it is important for the government to fully implement the 2012 Mental Health Act to increase awareness and access to quality mental health care.

The study's results revealed that the Kayayei with primary and Junior High School education were more likely to be stressed than the uneducated. This finding contradicts other studies arguing that more educated people experience lower rates of mental health problems than those with less education, presenting the notion that higher educational achievement is a protective factor against mental health disorders [44, 45]. The findings of the present study are likely because the education level of these Kayayei is relatively low, with a substantial number of them reported as having some level of primary education. Compared with the uneducated Kayayei who might be content with their current situation, those with some education might think they deserve better opportunities to further their education or get better employment opportunities, causing them mental health distress. The Kayayei, however, have limited knowledge and skills to manage mental health issues and do not have ready access to health and mental health care.

The study found that Kayayei, who rated the nature of their work as very difficult, had a higher likelihood of experiencing depression, stress, and anxiety. This finding is commensurate with previous findings that reported relationships between the nature of work and workers' mental health [46–48]. Even though employment, irrespective of the type, provides individuals with financial resources that can promote increased positive mental well-being, it may also contribute to the development of psychological distress if the conditions in which they perform are poor [49–52]. In this study, the mental health distress of the Kayayei could primarily be the extraneous job demands of their work as it is informal employment that involves lifting and carrying heavy loads from one place to another over long periods of time and distances. In most instances, the Kayayei, irrespective of their age must carry heavy loads bought by their clients on their heads to the desired destination. All these repetitive work routines are associated with musculoskeletal stress (pain) due to the weight of loads and falls that might also impact mental health due to the association between pain and mental health as indicated by Tantawy et al. [53]. It is recommended that vocational training opportunities be offered to the Kayayei so that they will be trained to acquire basic skills to be self-employed in areas that will not be harmful to their physical and mental health.

The present study's findings further indicated that the Kayayei maltreated by their clients were more likely to experience mental health distress (depression). Maltreatment and discrimination against migrant workers and their impact on mental health outcomes have also been reported in various other studies [54–56]. A study by Straiton et al. [57], using data from the living conditions survey among immigrants in Norway and with a sample size of 4399, found that perceived discrimination predicted significantly higher odds of mental health problems. A similar study by Schunck et al. also found that perceived discrimination predicted both mental and physical health concerns [58]. The findings are, thus, supported by various studies that suggest that maltreatment and discrimination against migrant workers is likely to have negative mental health consequences for them. It should also be noted that migrant women who engage in informal work might be more vulnerable to abuse and maltreatment because they often hold jobs for which there is little protection under social legislation [59, 60]. Furthermore, in most countries, women do not seem to have the same rights and opportunities for employment as men do. The women are, however, expected to take responsibility for the whole family's survival and look for sources of income no matter the circumstances [61, 62]. Society needs to consider the changing role of women in society, where women take on various responsibilities and treat them fairly in the workplace. It is essential to create awareness among the Kayayei community to know their rights and report abuse to law enforcement agencies. The Kayayei association can link its members to organisations, groups, agents, and other structures that provide appropriate support during challenging times to improve their mental health and general well-being.

The finding further indicated that participants who had accommodation were more likely to be stressed as compared to those without accommodation. This finding confirms an earlier study by Simning et al. [63] which found higher mental health distress levels among public housing residents. Another study by Simning et al. [64] among older adults in public housing also found higher levels of mental health distress among participants. Just like the present study, residents in public housing are mostly unemployed, may abuse drugs, and mostly had low income creating mental health distress. The socio-economic characteristics of the Kayayei are similar to the above studies, the Kayayei may feel housing insecurity because of the inconducive nature of accommodation as observed by Stahre et al. [65]. Low-income individuals worry about paying their rent and possibly becoming homeless. This insecurity can cause them psychological distress [65].

### Strengths and limitations of the study

This study demonstrated the presence of high levels of mental health distress (depression, anxiety, and stress) among internal female migrants (Kayayei) in Ghana. The study has demonstrated the magnitude of mental health concerns among a vulnerable segment of the population. The use of correlations and binary logistic regression has established the predictors of mental health distress among the study population. The use of a cross-sectional design study, where data were collected at a single point in time, was a limitation because it limited the interpretation of the findings. Due to this, the study was only able to find relationships between variables, but it was not able to make any suggestions regarding causation. However, because Agbogbloshie has the largest number of Kayayei in Ghana, the sample size was deemed adequate for the study and the findings can therefore be generalised to those in Accra.

## Conclusion

The majority of the Kayayei revealed high levels of mental health distress (depression, anxiety and Stress). This study recommends that the Ghana Labour Commission must extend the Labour Act 2003(Act 651) to cover the informal sector and create awareness among the Kayayei community to know their rights and report any abuse to law enforcement agencies. It is also recommended that the government, NGOs, and other benevolent organisations make an effort to train the Kayayei to attain alternative and sustained livelihoods that will not have negative impact on their mental as has been found in the current study. Finally, it is suggested that government should fully implement the 2012 Mental Health Act to increase awareness and access to quality mental health care, particularly among women.

## Data Availability

The datasets used and/or analysed for the current study is available from the corresponding author on reasonable request.
